# Neuroprotective Effects and Mechanisms of Arecoline Against H_2_O_2_-Induced Damage in SH-SY5Y Cells

**DOI:** 10.3390/ijms262110355

**Published:** 2025-10-24

**Authors:** Xiangfei Zhang, Jingwen Cui, Jing Sun, Fengzhong Wang, Bei Fan, Cong Lu

**Affiliations:** 1 Institute of Food Science and Technology, Chinese Academy of Agricultural Sciences, Beijing 100193, China; 82101235017@caas.cn (X.Z.); 18734021485@163.com (J.C.); ycsunjing2008@126.com (J.S.); wangfengzhong@sina.com (F.W.); 2 Institute of Food and Nutrition Development, Ministry of Agriculture and Rural Affairs, Beijing 100081, China

**Keywords:** arecoline, SH-SY5Y, oxidative stress, apoptosis, *Nrf2*/HO-1 pathway, mitochondrial apoptotic pathway

## Abstract

An overproduction of reactive oxygen species (ROS) creates oxidative stress that disrupts neuronal activity and contributes to the pathogenesis of neurodegenerative diseases. Arecoline, the predominant alkaloid component of *Areca catechu* L., is known for multiple biological activities, yet its involvement in neuronal oxidative injury has not been fully clarified. This study investigated arecoline’s effect on hydrogen peroxide (H_2_O_2_)-induced toxicity in SH-SY5Y human neuroblastoma cells (SH-SY5Y). Arecoline pretreatment significantly improved cell viability and preserved plasma membrane integrity, accompanied by reduced lipid peroxidation and restoration of cellular antioxidant enzyme activities. Moreover, arecoline maintained mitochondrial membrane potential and suppressed apoptotic progression. At the molecular level, Arecoline stimulated nuclear factor erythroid 2-related factor 2 (*Nrf2*) and heme oxygenase-1 (HO-1) protein expression, concurrently diminishing Kelch-like ECH-associated protein 1 (*Keap1*) levels. In parallel, it altered the apoptosis profile by increasing B-cell lymphoma 2 (*Bcl2*) levels and decreasing Bcl-2-associated X protein (*Bax*) and total cysteine aspartate protease-3 (Caspase-3) protein expression. Collectively, the findings suggest that arecoline safeguards neurons against oxidative stress by simultaneously activating antioxidant defenses and restraining apoptosis. This study adds novel molecular evidence supporting the potential neuroprotective relevance of arecoline in oxidative stress-related neuropathology.

## 1. Introduction

Oxidative stress is broadly acknowledged as a core driver of neuronal damage and the advancement of neurodegenerative disorders [1,2]. An overabundance of reactive oxygen species (ROS) wreaks havoc on cellular components—oxidizing lipids, degrading proteins, and mutilating genetic material. This oxidative onslaught impairs mitochondrial efficiency and triggers programmed cell death pathways, culminating in the irreversible destruction of neurons [3,4]. Clinical and experimental studies consistently demonstrate that oxidative imbalance contributes to cognitive decline [5,6], emotional dysregulation [7,8], and progressive neurodegeneration [9], highlighting the restoration of redox homeostasis as a critical therapeutic strategy. Therefore, natural bioactive compounds with antioxidative and neuroprotective potential have attracted considerable attention as promising candidates for mitigating oxidative stress-related neuronal injury [10,11].

Among natural sources of neuroactive compounds, *Areca catechu* L. (areca nut) is traditionally consumed as a masticatory and recognized in Chinese medicine as one of the “Four Southern Medicines” due to its diverse pharmacological properties [12]. Arecoline, its major alkaloid, has attracted increasing attention for multiple neuropharmacological activities. Experimental studies have shown that arecoline enhances cognitive performance in disease models [13], while clinical investigations in Alzheimer’s patients further reported improvements in memory functions, including verbal recall and picture recognition, following arecoline administration [14,15]. Moreover, arecoline demonstrates antidepressant properties in rodent studies, decreasing immobility during forced swim and tail suspension tests [16]. Clinical studies further support its relevance to depression, with the cholinergic rapid eye movement (REM) induction test revealing altered muscarinic receptor sensitivity in patients [17]. Both preclinical and clinical trials demonstrate anxiolytic effects, with arecoline reducing anxiety-related behaviors through modulation of neurotransmitter systems such as acetylcholine, dopamine, and serotonin [18,19]. Previous research has predominantly addressed the cholinergic and behavioral effects of arecoline, whereas evidence delineating its direct antioxidant and anti-apoptotic activities remains scarce. To date, no study has comprehensively demonstrated whether arecoline can mitigate oxidative damage in neuronal cells through intrinsic mechanisms, nor elucidated its potential regulation of the Nrf2/HO-1-mediated antioxidant defense and the Bcl-2/Bax/total Caspase-3-dependent apoptotic pathways under oxidative stress conditions.

Derived from a human neuroblastoma, SH-SY5Y cells exhibit neuron-like morphology and express multiple neuron-specific proteins, including neurofilaments and catecholaminergic enzymes such as tyrosine hydroxylase and dopamine-β-hydroxylase [20,21]. These cells can be induced to differentiate into mature neuron-like phenotypes, display neurotransmitter synthesis and release, and show high sensitivity to oxidative and mitochondrial stress [22]. Owing to these human-derived neuronal properties and reproducible growth characteristics, SH-SY5Y cells have become a widely accepted system for investigating the molecular mechanisms of oxidative injury and evaluating the neuroprotective efficacy of natural compounds under controlled experimental conditions [23,24].

To fully elucidate the molecular basis of arecoline’s neuroprotective potential, we employed an H_2_O_2_-induced SH-SY5Y neurotoxicity model and assessed its effects on cell viability, oxidative stress indicators, mitochondrial membrane potential, and apoptosis. Particular attention was given to the antioxidant Nrf2/HO-1 pathway and the apoptotic Bcl-2/Bax/total Caspase-3 signaling cascades. The results provide new evidence that arecoline exerts antioxidative and anti-apoptotic effects, thereby extending its pharmacological relevance beyond classical cholinergic regulation.

## 2. Results

### 2.1. Establishment of the H_2_O_2_-Induced Oxidative Injury Model and Evaluation of Arecoline Cytotoxicity in SH-SY5Y Cells

**Figure 1:**
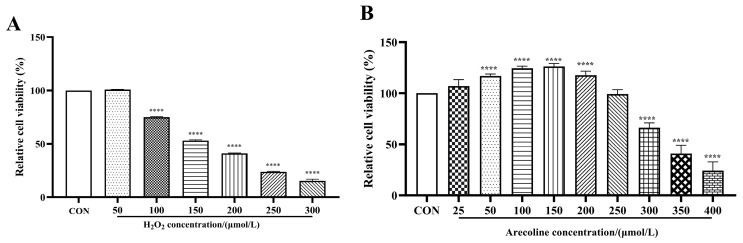
Effects of H_2_O_2_ and arecoline on SH-SY5Y cell viability. (**A**) Cells were treated with different concentrations of H_2_O_2_ (50–300 μmol/L) for 24 h to determine the optimal oxidative injury dose. (**B**) Cells were exposed to arecoline (25–400 μmol/L) for 24 h to assess its dose–response effects on viability. Results are shown as mean ± SEM (*n* = 5). **** *p* < 0.0001 vs. control. The CON group was defined as 100% for normalization; consequently, no error bars are shown for this group.

SH-SY5Y cells were exposed to increasing concentrations of H_2_O_2_ (50–300 μmol/L) for 24 h, and cell viability was assessed using the CCK-8 assay (Figure 1A). Cell survival decreased in a concentration-dependent manner, with significant reductions observed from 100 μmol/L (*p* < 0.0001) and a pronounced decline at higher doses (200–300 μmol/L). Exposure to 150 μmol/L H_2_O_2_ reduced cell viability to approximately 50%, representing a suitable condition that induced evident oxidative injury while maintaining sufficient cell survival. Therefore, 150 μmol/L H_2_O_2_ was selected for subsequent experiments.

The effects of arecoline on SH-SY5Y cell viability were evaluated using the CCK-8 assay. The arecoline concentrations (25–400 μmol/L) were selected based on preliminary screening experiments designed to identify a range that does not cause significant cytotoxicity in SH-SY5Y cells. As shown in Figure 1B, arecoline exhibited a biphasic (hormetic) response, with a slight increase in cell viability at low concentrations (25–150 μmol/L) and marked cytotoxicity above 200 μmol/L. Because the dose–response curve exhibited a biphasic pattern rather than a monotonic decline, IC_50_ fitting was not applicable. Therefore, the concentration range that maintained ≥80% cell viability (35–140 μmol/L) was selected as non-cytotoxic for subsequent experiments.

### 2.2. Effects of Arecoline Against H_2_O_2_-Induced Cytotoxicity in SH-SY5Y Cells

**Figure 2:**
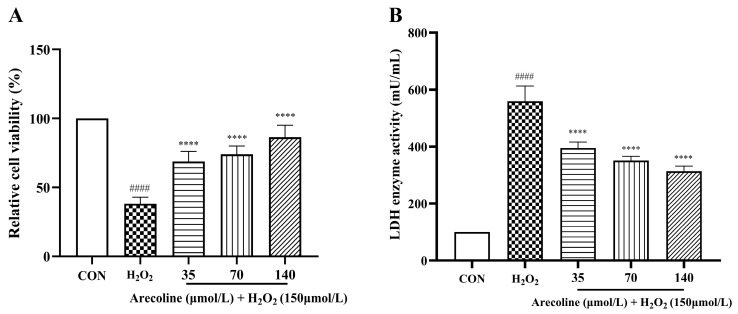
Effects of arecoline on H_2_O_2_-induced cytotoxicity in SH-SY5Y cells. (**A**) Cell viability. (**B**) Lactate dehydrogenase (LDH) release in culture supernatants. Results are shown as mean ± SEM (*n* = 5). #### *p* < 0.0001 vs. control; **** *p* < 0.0001 vs. model. The CON group was defined as 100% for normalization; consequently, no error bars are shown for this group.

Exposure to 150 μmol/L H_2_O_2_ for 24 h markedly reduced SH-SY5Y cell viability compared with the control group (*p* < 0.0001; Figure 2A), confirming successful establishment of the oxidative stress model. Pretreatment with arecoline (35–140 μmol/L) significantly increased cell viability under oxidative conditions (*p* < 0.0001), indicating a concentration-dependent cytoprotective effect. Importantly, arecoline alone at the tested concentrations did not induce cytotoxicity, which is consistent with the viability data shown in Figure 1B.

Consistently, LDH activity in the culture medium was substantially elevated after H_2_O_2_ treatment relative to control cells (*p* < 0.0001; Figure 2B), reflecting membrane damage. Arecoline pretreatment at all tested doses significantly suppressed LDH release (*p* < 0.0001), suggesting improved membrane integrity. Together, these findings demonstrate that arecoline mitigates H_2_O_2_-induced cytotoxicity by enhancing cell survival and reducing oxidative membrane injury.

### 2.3. Effects of Arecoline on Oxidative Stress Markers in H_2_O_2_-Induced SH-SY5Y Cells

**Figure 3:**
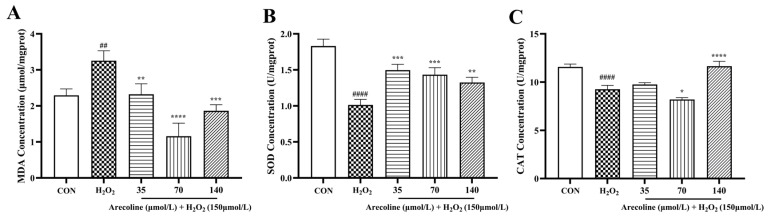
Effects of arecoline on oxidative stress markers in H_2_O_2_-induced SH-SY5Y cells. (**A**) Malondialdehyde (MDA) levels. (**B**) Superoxide dismutase (SOD) levels. (**C**) Catalase (CAT) levels. Results are shown as mean ± SEM (*n* = 5). ## *p* < 0.01, #### *p* < 0.0001 vs. control; * *p* < 0.05, ** *p* < 0.01, *** *p* < 0.001, **** *p* < 0.0001 vs. model.

As shown in Figure 3A, H_2_O_2_ treatment markedly increased intracellular malondialdehyde (MDA) content compared with the control group (*p* < 0.01), indicating elevated lipid peroxidation. Pretreatment with arecoline (35–140 μmol/L) significantly suppressed this rise (*p* < 0.01–0.0001), suggesting inhibition of oxidative membrane damage.

Conversely, superoxide dismutase (SOD) activity was significantly reduced by H_2_O_2_ exposure (*p* < 0.0001; Figure 3B), while all tested arecoline concentrations markedly restored SOD levels (*p* < 0.001), reflecting recovery of enzymatic antioxidant defense. Similarly, catalase (CAT) activity was decreased in the H_2_O_2_ group (*p* < 0.0001; Figure 3C), but were significantly enhanced by arecoline pretreatment, especially at 70 and 140 μmol/L (*p* < 0.05 and *p* < 0.0001).

Collectively, these findings demonstrate that arecoline alleviates H_2_O_2_-induced oxidative stress in SH-SY5Y cells by reducing lipid peroxidation and restoring antioxidant enzyme activities.

### 2.4. Effects of Arecoline on MMP and Apoptosis in H_2_O_2_-Induced SH-SY5Y Cells

**Figure 4:**
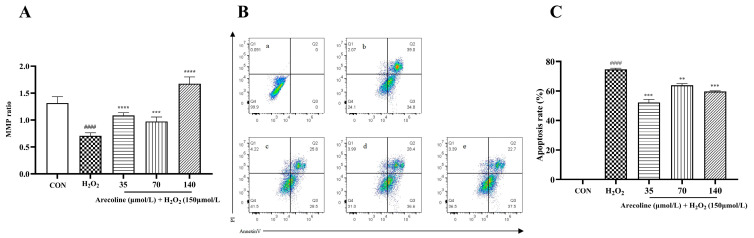
Effects of arecoline on mitochondrial membrane potential (MMP) and apoptosis in H_2_O_2_-induced SH-SY5Y cells. (**A**) Quantification of MMP ratio. (**B**) Representative flow cytometry plots showing apoptotic distribution. a (CON), b (H_2_O_2_), c (arecoline 35 μmol/L), d (arecoline 70 μmol/L), e (arecoline 140 μmol/L). (**C**) Apoptotic ratio statistics. Results are shown as mean ± SEM (*n* = 5). #### *p* < 0.0001 vs. control; ** *p* < 0.01, *** *p* < 0.001, **** *p* < 0.0001 vs. model.

As shown in Figure 4A, exposure to H_2_O_2_ (150 µmol/L) markedly reduced the mitochondrial membrane potential (MMP) compared with the control group (**** *p* < 0.0001). Pretreatment with arecoline (35–140 µmol/L) significantly restored the MMP in a concentration-dependent manner (*** *p* < 0.001 to **** *p* < 0.0001).

Flow cytometric analysis using Annexin V–FITC/PI staining (Figure 4B) revealed a pronounced increase in apoptotic cells after H_2_O_2_ exposure (**** *p* < 0.0001). Quantitative analysis (Figure 4C) showed that arecoline pretreatment at all tested concentrations significantly decreased the apoptosis rate (** *p* < 0.01 to *** *p* < 0.001).

These data demonstrate that arecoline effectively mitigates mitochondrial depolarization and reduces apoptosis in SH-SY5Y cells under H_2_O_2_-induced oxidative stress.

### 2.5. Effects of Arecoline on Nrf2/HO-1/Keap1 Signaling Pathways in H_2_O_2_-Induced SH-SY5Y Cells

**Figure 5:**
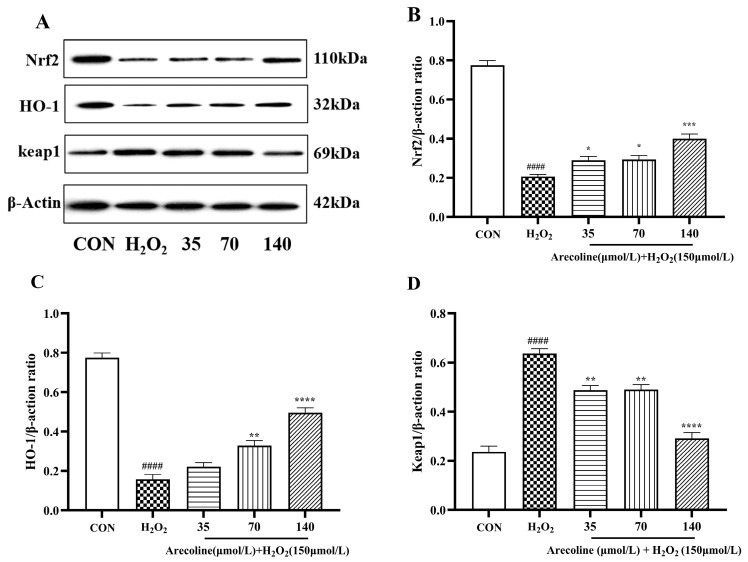
Modulatory role of arecoline in the *Nrf2*/HO-1/*Keap1* pathway in H_2_O_2_-induced SH-SY5Y cells. (**A**) Western blot bands. (**B**) Nrf2/β-actin. (**C**) HO-1/β-actin. (**D**) Keap1/β-actin. Results are shown as mean ± SEM (*n* = 3). #### *p* < 0.0001 vs. control; * *p* < 0.05, ** *p* < 0.01, *** *p* < 0.001, **** *p* < 0.0001 vs. model.

Western blot analysis showed that oxidative stress markedly disturbed the expression pattern of key antioxidant signaling proteins (Figure 5A–D). Exposure to H_2_O_2_ led to a pronounced decline in *Nrf2* and HO-1 protein levels (*p* < 0.0001), accompanied by a significant upregulation of *Keap1* (*p* < 0.0001) compared with the control group. These changes suggest that H_2_O_2_ impairs the intrinsic antioxidant defense system by inhibiting *Nrf2* activation and promoting *Keap1* accumulation, thereby disrupting cellular redox equilibrium.

Pretreatment with arecoline effectively counteracted these alterations. *Nrf2* and HO-1 expression increased in a clear dose-dependent manner (*p* < 0.05–0.0001), while *Keap1* expression declined correspondingly. The strongest regulatory effects were observed at 140 μmol/L arecoline, indicating robust activation of the *Nrf2*/HO-1 signaling axis and suppression of Keap1-mediated inhibition.

Together, these findings indicate that arecoline restores oxidative balance, which is associated with activation of the *Nrf2*/HO-1 pathway and downregulation of *Keap1*, potentially contributing to its protective effects against oxidative stress-induced neuronal injury.

### 2.6. Effects of Arecoline on Bcl-2/Bax/Total Caspase-3 Signaling Pathways in H_2_O_2_-Induced SH-SY5Y Cells

**Figure 6:**
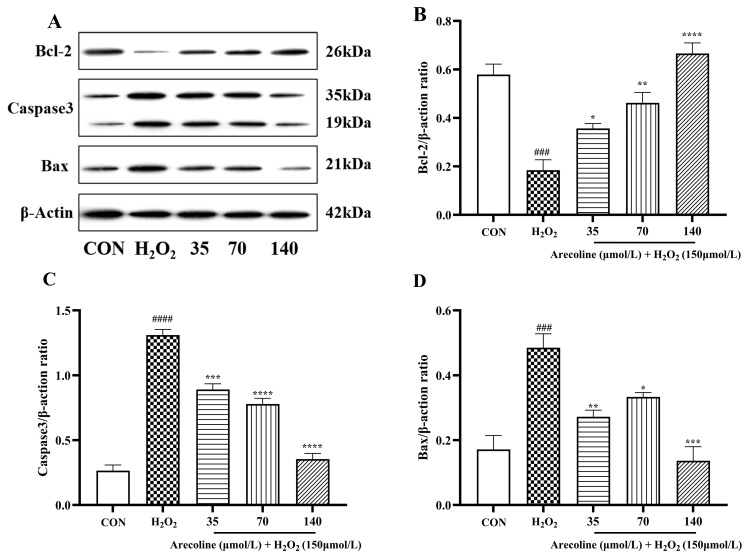
Modulatory role of arecoline in the Bcl-2/Bax/total Caspase-3 pathway in H_2_O_2_-induced SH-SY5Y cells. (**A**) Western blot bands. (**B**) Bcl-2/β-actin. (**C**) Total Caspase-3/β-actin. (**D**) Bax/β-actin. Results are shown as mean ± SEM (*n* = 3). ### *p* < 0.001, #### *p* < 0.0001 vs. control; * *p* < 0.05, ** *p* < 0.01, *** *p* < 0.001, **** *p* < 0.0001 vs. model.

Western blot analysis revealed that oxidative stress significantly altered the expression of apoptosis-related proteins in SH-SY5Y cells (Figure 6A–D). Treatment with H_2_O_2_ markedly reduced the expression of the anti-apoptotic protein Bcl-2 (*p* < 0.0001; Figure 6A,B), while substantially increasing the levels of the pro-apoptotic proteins total Caspase-3 and Bax (*p* < 0.0001; Figure 6C,D). These changes confirm that oxidative stress strongly triggers apoptotic signaling in neuronal cells.

Pretreatment with arecoline effectively counteracted these effects in a dose-dependent manner. Bcl-2 expression was progressively restored (*p* < 0.05–0.0001), accompanied by a significant reduction in total Caspase-3 and Bax levels (*p* < 0.01–0.0001). The most prominent regulatory effect was observed at 140 μmol/L arecoline, where the balance between pro- and anti-apoptotic proteins shifted markedly toward cell survival.

Collectively, these findings indicate that arecoline protects SH-SY5Y cells from H_2_O_2_-induced apoptosis in association with re-establishment of the Bcl-2/Bax/total Caspase-3 pathway, thereby promoting neuronal resilience under oxidative stress conditions.

## 3. Discussion

Although the present study highlights the neuroprotective and anti-inflammatory properties of arecoline, it is important to acknowledge its well-documented toxic and carcinogenic potential. Arecoline, the principal alkaloid of *Areca catechu* (areca nut), has been classified by the International Agency for Research on Cancer (IARC) as a Group 2B carcinogen due to its established association with oral submucous fibrosis and oral carcinogenesis upon chronic exposure [25,26,27]. Notably, its biological effects are context dependent—a phenomenon often referred to as the “arecoline paradox.” Low or transient concentrations may activate adaptive cellular defense pathways and exert neuroprotective actions in neuronal systems, whereas prolonged or high-dose exposure can lead to oxidative imbalance, apoptosis, and genotoxicity, particularly in oral epithelial cells [28]. Therefore, the pharmacological potential of arecoline should be interpreted cautiously and confined to safe, non-toxic experimental ranges.

H_2_O_2_ is often used in vitro as a stable generator of ROS, as it can readily penetrate cell membranes, disturb intracellular redox balance, and trigger apoptotic signaling [29,30,31]. The SH-SY5Y cell line, which exhibits catecholaminergic activity and high sensitivity to oxidative insults, is widely employed as a neuronal model [23,24,32]. Accordingly, the H_2_O_2_-induced SH-SY5Y system is a well-established platform for studying oxidative injury and evaluating candidate neuroprotectants [33,34]. In our study, exposure to H_2_O_2_ produced characteristic oxidative damage, thereby validating the reliability of the cellular model.

In this study, arecoline exhibited a biphasic (hormetic) dose–response pattern, characterized by a mild increase in cell viability at low concentrations and a marked decrease at higher doses. Although SH-SY5Y cells originate from a neuroblastoma lineage, numerous studies have demonstrated that these cells can be functionally differentiated into neuron-like, low-proliferative phenotypes under standard culture conditions [32,35]. Therefore, the mild increase in cell viability observed at low arecoline concentrations may reflect a transient adaptive response to mild physiological stress, consistent with reports that arecoline modulates neuronal metabolism and monoamine oxidase A (MAO-A) expression in SH-SY5Y cells [36]. Such a pattern is indicative of hormetic activation of cellular defense mechanisms [37].

LDH is a cytosolic enzyme released during membrane damage and is commonly used as an indicator of cellular injury [38]. In line with previous studies using H_2_O_2_ to model oxidative stress, we observed a marked decrease in SH-SY5Y cell viability together with elevated LDH release, reflecting impaired metabolic activity and loss of membrane integrity [39,40]. Pretreatment with arecoline significantly restored cell viability and reduced LDH leakage, indicating that it effectively counteracts early oxidative damage and confers cytoprotective effects at the cellular level.

Beyond early indicators of cell damage, we next assessed oxidative stress parameters to further characterize the protective role of arecoline. Consistent with the oxidative stress paradigm, H_2_O_2_ exposure markedly increased MDA levels while reducing the activities of key antioxidant enzymes, SOD and CAT [41,42,43]. These changes align with the well-established role of enzymatic antioxidants in maintaining redox balance, where SOD dismutates superoxide radicals and CAT decomposes H_2_O_2_ [44,45]. Pretreatment with arecoline reversed these alterations, lowering MDA accumulation and restoring antioxidant enzyme activities, thereby demonstrating its antioxidative efficacy. Since SOD and CAT activities are tightly regulated by upstream transcriptional programs, we further assessed the involvement of the *Nrf2*/HO-1 pathway. *Nrf2* is a master regulator of redox homeostasis that dissociates from Keap1 under oxidative challenge and translocates into the nucleus to induce antioxidant genes such as HO-1 [46,47,48]. Previous work has shown that H_2_O_2_ suppresses *Nrf2*/HO-1 activity, thereby exacerbating neuronal vulnerability [40,49], arecoline markedly enhanced *Nrf2* and HO-1 expression while downregulating *Keap1*. This dual action both reduced oxidative stress directly and strengthened endogenous defense systems, highlighting a novel antioxidative mechanism for arecoline beyond its classical cholinergic profile.

Since oxidative stress is a well-known trigger of mitochondrial dysfunction and apoptosis [50,51], we further assessed whether arecoline could protect SH-SY5Y cells from H_2_O_2_-induced apoptotic injury. MMP disruption is a key hallmark of apoptosis and is commonly observed under oxidative stress [52]. Consistent with the previous reports [53,54], we observed that H_2_O_2_ induced a collapse of MMP, expansion of apoptotic cell populations, and upregulation of pro-apoptotic proteins. Remarkably, arecoline preserved mitochondrial polarization, reduced apoptosis, and stabilized energy status, suggesting direct protection of mitochondrial function. At the molecular level, mitochondrial apoptosis is chiefly governed by the Bcl-2 protein family together with the caspase signaling cascade. Anti-apoptotic members, exemplified by Bcl-2, contribute to the preservation of mitochondrial membrane stability, whereas pro-apoptotic proteins such as Bax facilitate cytochrome *c* efflux and trigger the activation of executioner caspases, notably total caspase-3 [55,56]. Disturbance of the equilibrium between survival-promoting and death-promoting factors is recognized as a defining feature of oxidative stress-driven neuronal damage. In the current work, arecoline was shown to influence this regulatory network by upregulating Bcl-2 expression while concomitantly reducing Bax and total caspase-3, thereby re-establishing the balance between prosurvival and proapoptotic signals. By modulating mitochondrial apoptosis, arecoline appears to offer an additional protective mechanism, suggesting its dual capacity to attenuate oxidative stress and suppress downstream apoptotic cascades.

Collectively, our results provide the first evidence that arecoline confers protection against H_2_O_2_-induced neuronal injury by simultaneously enhancing antioxidative defenses and suppressing apoptotic signaling. The protective effects observed were associated with activation of the *Nrf2*/HO-1 antioxidant pathway and modulation of the Bcl-2/Bax/total Caspase-3 apoptotic cascade, suggesting that these pathways may cooperatively contribute to enhanced neuronal survival under oxidative stress. Considering that redox imbalance, mitochondrial impairment, and programmed cell death are well-established contributors to the pathogenesis of neurodegenerative disorders such as Alzheimer’s and Parkinson’s disease, these findings not only expand the pharmacological characterization of arecoline but also underscore its potential as a neuroprotective agent. Overall, our findings highlight arecoline as a neuroprotective compound that acts through complementary antioxidative and anti-apoptotic mechanisms, warranting continued investigation into its therapeutic applicability in neurodegeneration.

In summary, this study demonstrates that arecoline protects SH-SY5Y neuronal cells from H_2_O_2_-induced oxidative stress and apoptosis through activation of the *Nrf2*/HO-1 pathway and modulation of the Bcl-2/Bax/total Caspase-3 cascade. Because only total Caspase-3 was assessed, this change should be interpreted as an association with reduced apoptotic signaling rather than direct evidence of inhibited Caspase-3 activation. This work primarily focused on oxidative stress, apoptosis, and antioxidant responses at the protein and functional levels. Further investigations incorporating quantitative gene expression profiling, ROS measurement, and GSH quantification could provide deeper mechanistic insight. In addition, experiments using pharmacological inhibitors such as ML385 or genetic silencing approaches (*Nrf2* or *Bcl2* siRNA) will be necessary to verify whether activation of these pathways is essential for the observed neuroprotective effects.

## 4. Materials and Methods

### 4.1. Materials

Arecoline (molecular weight 155.20 g/mol; CAS No. 63-75-2; purity ≥ 98%) was obtained from Shanghai Yuanye Bio-Technology Co., Ltd. (Shanghai, China), and SH-SY5Y human neuroblastoma cells with culture medium were provided by Wuhan Shine Biological Technology Co., Ltd. (Wuhan, China) H_2_O_2_, dimethyl sulfoxide, the Cell Counting Kit-8, and detection kits for lactate dehydrogenase (LDH), bicinchoninic acid (BCA), SOD, MDA, CAT, and mitochondrial membrane potential (MMP) were from Beyotime Biotechnology (Shanghai, China). Antibodies specific for Nrf2, HO-1, Keap1, Bcl-2, Bax, total Caspase-3, and β-actin were sourced from Proteintech Group, Inc. (Wuhan, China); Abcam plc (Cambridge, UK); Cell Signaling Technology, Inc. (Danvers, MA, USA); and Solarbio Science & Technology Co., Ltd. (Beijing, China).

The main laboratory instruments used in this study included a CO_2_ incubator (Thermo 371; Thermo Fisher Scientific, Waltham, MA, USA), refrigerator (Midea Biomedical Technology Co., Ltd., Hefei, China), water bath (HWS-24; Shanghai Yiheng, Shanghai, China), Milli-Q ultrapure water system (Millipore, Burlington, MA, USA), inverted microscope (IX71; Olympus, Tokyo, Japan), high-speed centrifuge (KH23A; Hunan Herexi, Changsha, China), flow cytometer (CytoFLEX; Beckman Coulter, Brea, CA, USA), microplate reader (SpectraMax iD3; Molecular Devices, San Jose, CA, USA), scanner (V370; Epson, Nagano, Japan), and refrigerated tabletop centrifuges (TG-16; Xiangyi, Changsha, China; and 5810R; Eppendorf, Hamburg, Germany).

### 4.2. Cell Culture Conditions and Experimental Protocol

SH-SY5Y cells were cultured in Dulbecco’s Modified Eagle Medium (DMEM) supplemented with 10% fetal bovine serum (FBS) and 1% penicillin–streptomycin (100 U/mL and 100 μg/mL, respectively) under standard conditions (37 °C, 5% CO_2_). Cells between passages 5 and 20 in the logarithmic growth phase were used for all experiments.

Arecoline was dissolved in dimethyl sulfoxide (DMSO) and diluted with serum-free DMEM to the indicated concentrations, with the final DMSO concentration maintained below 0.1% (*v*/*v*). Hydrogen peroxide (H_2_O_2_) was freshly diluted in DMEM before use.

The experimental workflow comprised two stages. First, the cytotoxicity of arecoline (25–400 μmol/L, 24 h) was evaluated using the CCK-8 assay to determine the non-cytotoxic concentration range. Based on these data (Figure 1), 35–140 μmol/L were selected as safe concentrations for subsequent assays.

Oxidative stress injury was then induced with H_2_O_2_ (150 μmol/L, 24 h), and the protective effects of arecoline were examined under the following conditions: (1) control (untreated cells), (2) H_2_O_2_ (150 μmol/L for 24 h), (3) arecoline alone (35, 70, or 140 μmol/L for 24 h), and (4) arecoline + H_2_O_2_ (arecoline pretreatment for 24 h followed by 150 μmol/L H_2_O_2_ for another 24 h).

Cell viability, LDH release, oxidative stress markers, mitochondrial membrane potential, and apoptosis-related protein expression were assessed to evaluate the protective role of arecoline against H_2_O_2_-induced neuronal injury. All functional and biochemical assays were performed with five independent biological replicates (*n* = 5), and Western blot analyses were conducted in three independent experiments (*n* = 3). Technical replicates were averaged within each biological replicate prior to statistical analysis.

### 4.3. Establishment of the H_2_O_2_-Induced Cell Injury Model and Cytotoxicity of Arecoline

Log-phase SH-SY5Y cells were seeded in 96-well plates at 5 × 10^5^ cells/mL, dispensing 100 µL per well for each experimental condition. This corresponds to the optimal culture density reported for SH-SY5Y cells [57]. This density was appropriate for 24 h viability and oxidative stress assays. The groups consisted of: untreated controls with fresh DMEM; H_2_O_2_ exposure (50–300 μmol/L, 24 h); and arecoline exposure (25–400 μmol/L, 24 h). Following the incubation period, 10 μL of CCK-8 reagent was added to each well, and plates were further maintained as instructed. Optical density was finally recorded at 450 nm using a microplate reader.

The concentrations of H_2_O_2_ (50–300 μmol/L) were selected according to previous studies that commonly employed this range to induce oxidative stress in SH-SY5Y neuronal cells [58,59]. The arecoline concentrations (25–400 μmol/L) were chosen based on reported salivary levels in betel-quid chewers (40–400 μmol/L) and in vitro studies using comparable doses to evaluate cellular stress and toxicity [60,61].

### 4.4. Evaluation of Protective Effects of Arecoline on H_2_O_2_-Induced Cytotoxicity

Cells were plated following Section 4.3. SH-SY5Y cells were pretreated with arecoline (35, 70, or 140 μmol/L, 24 h) followed by H_2_O_2_ exposure (150 μmol/L, 24 h), cell viability analysis was carried out according to Section 4.3, and LDH release was additionally determined to evaluate membrane integrity and cellular injury.

### 4.5. Measurement of Oxidative Stress Markers (MDA, SOD, and CAT)

SH-SY5Y cells at the logarithmic growth stage were distributed into 6-well plates (6 × 10^5^ cells/mL, 2 mL per well), with five wells arranged for each condition. Protein levels of the collected samples were first determined using the BCA approach, and these values were applied to normalize subsequent enzyme assays. MDA, SOD, and CAT were measured with commercial kits (Beyotime, Shanghai, China).

### 4.6. Assessment of MMP and Apoptosis

Cells were plated following Section 4.5. Apoptosis was assessed via Annexin V-FITC/PI staining, while JC-1 probe flow cytometry (Beckman Coulter CytoFLEX, Beckman Coulter Inc., Brea, CA, USA) measured MMP.

### 4.7. Protein Expression Analysis by Western Blot

Cells were plated following Section 4.5. SH-SY5Y cells were subjected to lysis in RIPA buffer that was fortified with protease inhibitors. Protein levels were assessed via the BCA protocol. A 30-μg protein sample was resolved via SDS-PAGE, blotted onto PVDF membranes, and blocked using 5% BSA. The membranes were left to incubate overnight at a chilly 4 degrees Celsius, during which time they were exposed to primary antibodies. Following washing, the membranes were subjected to secondary antibodies conjugated with HRP. Protein bands were visualized using an enhanced chemiluminescence (ECL) substrate, and band intensities were quantified with Image-Pro software (version 6.0, Media Cybernetics, Rockville, MD, USA). The information of primary antibodies used for Western blotting is summarized in Table 1.

### 4.8. Statistical Analysis

Our research utilized SPSS 26.0 for the data crunching and GraphPad Prism 8.0 for the plotting of graphs. To gauge the strength of our Western blot results, we leaned on Image-Pro software for the numbers. We evaluated variations between several groups with a one-way ANOVA, and we confirmed individual pairings with the LSD post hoc test. We deemed anything below a *p*-value of 0.05 as a solid statistical find.

## 5. Conclusions

In summary, this study demonstrates that arecoline protects SH-SY5Y neuronal cells from H_2_O_2_-induced oxidative stress and apoptosis. The protective effects are mediated through activation of the Nrf2/HO-1 antioxidant pathway and regulation of the Bcl-2/Bax/Caspase-3 apoptotic cascade, which together help maintain mitochondrial integrity and enhance cell survival. These results expand current understanding of arecoline as a potential neuroprotective agent and highlight its relevance for the prevention of oxidative injury in neuronal systems. Further studies, including in vivo validation and pathway inhibition assays, are required to confirm the underlying mechanisms and ensure experimental safety.

## Figures and Tables

**Table 1:** Information on primary antibodies used for Western blotting and their dilution ratios.

Antibody Target	Primary Antibody	Source	Dilution Ratio	Molecular Size
1	HO-1	Rabbit	1:1000	33 kDa
2	Bcl-2	Rabbit	1:2000	26 kDa
3	Bax	Rabbit	1:2000	21 kDa
4	Nrf2	Rabbit	1:1000	110 kDa
5	Caspase3	Rabbit	1:2000	35/19 kDa
6	Keap1	Rabbit	1:2000	69 kDa
7	β-actin	Rabbit	1:1000	42 kDa

## Data Availability

The original contributions presented in the study are included in the article, further inquiries can be directed to the corresponding authors.

## Abbreviations

The following abbreviations are used in this manuscript:

SH-SY5Y	Human neuroblastoma cell line
H_2_O_2_	Hydrogen peroxide
LDH	Lactate dehydrogenase
BCA	Bicinchoninic acid
MDA	Malondialdehyde
SOD	Superoxide dismutase
CAT	Catalase
MMP	Mitochondrial membrane potential
Nrf2	Nuclear factor erythroid 2-related factor 2
HO-1	Heme oxygenase-1
Keap1	Kelch-like ECH-associated protein 1
Bcl-2	B-cell lymphoma 2
Bax	Bcl-2-associated X protein
Caspase-3	Cysteine-aspartic acid protease 3
CCK-8	Cell Counting Kit-8
FBS	Fetal bovine serum
DMEM	Dulbecco’s Modified Eagle Medium
PVDF	Polyvinylidene fluoride
SDS-PAGE	Sodium dodecyl sulfate–polyacrylamide gel electrophoresis
HRP	Horseradish peroxidase
ECL	Enhanced chemiluminescence
